# The Oxidative Stress: Origin and Role in Aging and Diseases

**DOI:** 10.3390/antiox15050597

**Published:** 2026-05-08

**Authors:** Gabriella D’Orazi, Luca Neroni, Alessandra Verdina

**Affiliations:** 1 Departmental Faculty of Medicine, UniCamillus-Saint Camillus International University of Health Sciences, 00131 Rome, Italy; gabriella.dorazi@unicamillus.org; 2 Unit of Cellular Networks and Molecular Therapeutic Targets, Department of Research and Advanced Technologies, IRCCS Regina Elena National Cancer Institute, 00144 Rome, Italy; 3 Unit of Radiology and Diagnostic Imaging, Department of Clinical and Dermatological Research, IRCCS San Gallicano Dermatological Institute, 00144 Rome, Italy; luca.neroni@ifo.it

**Keywords:** reactive oxygen species (ROS), oxidative stress, redox balance, antioxidants, cellular damage, chronic inflammatory diseases, cancer, aging

## Abstract

Reactive oxygen species (ROS) play a dual role in biological systems: at low-to-moderate concentrations, they function as essential signaling molecules involved in processes such as cell proliferation, differentiation, and immune defense. However, excessive ROS production or impaired antioxidant defenses lead to oxidative stress, which can damage lipids, proteins, and DNA, contributing to the development and progression of various pathological conditions including cancer, cardiovascular diseases, neurodegenerative disorders, and aging. Understanding and managing oxidative stress is critical to promoting health and preventing chronic conditions. This review explores the growing body of literature surrounding this multifaceted and increasingly important area of study.

## 1. Introduction

Under normal physiological conditions, a carefully modulated redox balance is maintained by the interplay between pro-oxidant and antioxidant systems. The pro-oxidant system comprises reactive oxygen species (ROS), including oxygen-derived radicals such as the superoxide anion (O_2_^−^), hydroxyl radical (•OH), and hydrogen peroxide (H_2_O_2_). The antioxidant system includes enzymatic components such as catalase, superoxide dismutase (SOD), and glutathione peroxidase, as well as non-enzymatic molecules like glutathione, vitamin C (ascorbic acid), and vitamin E (tocopherol) (Table 1) [1,2,3,4].

**Figure 1:**
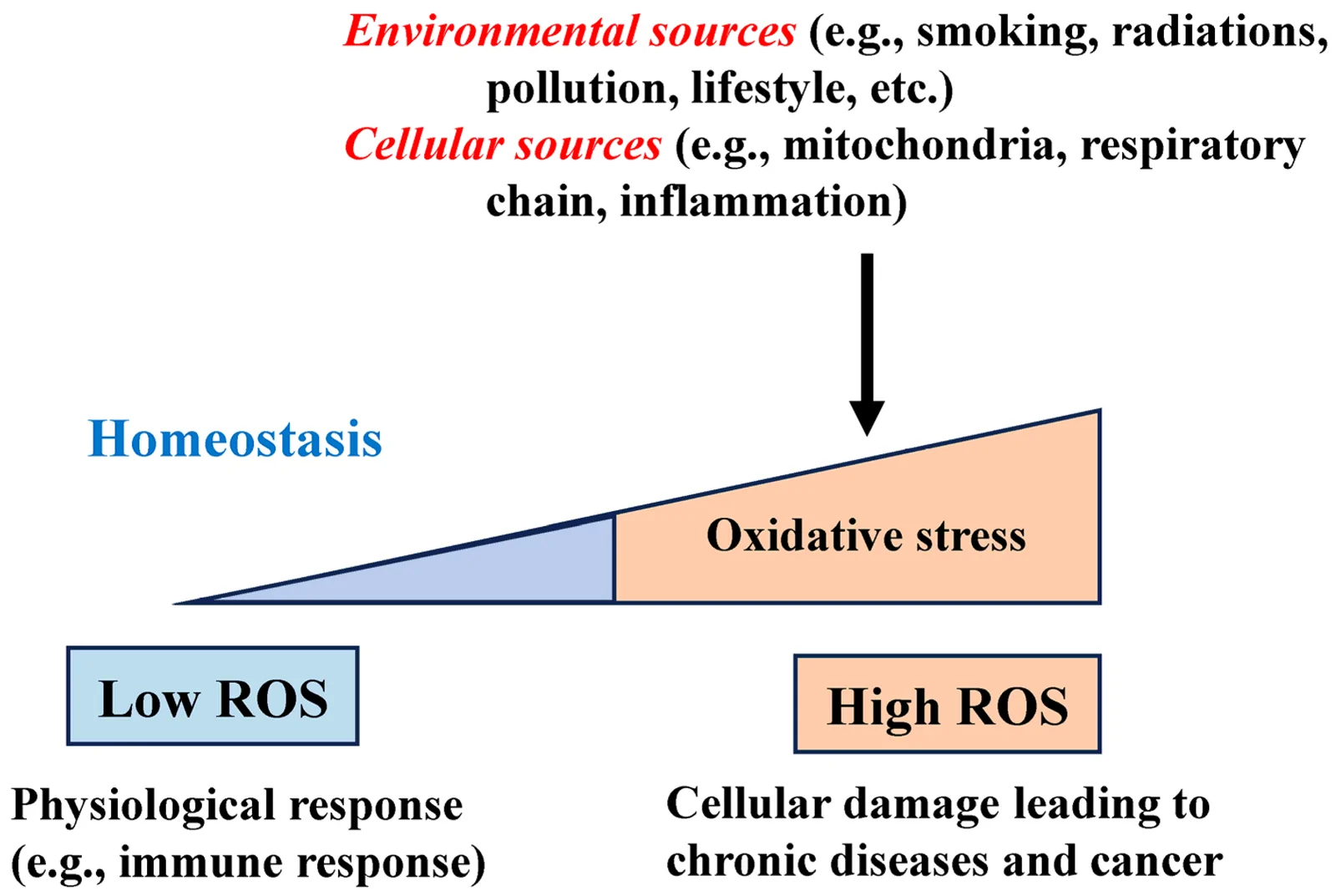
Schematic figure showing the Redox balance in physiological and pathological conditions. In the homeostatic condition, low ROS levels play a role in physiological processes. When high production of ROS, from environmental or cellular sources, overcomes the antioxidant activity, oxidative stress occurs. High ROS levels lead to cellular damage contributing to the development of chronic diseases.

The redox balance is a defense system that not only prevents cellular damage but also allows ROS to serve essential physiological roles. At low concentrations, ROS act as key signaling molecules involved in various processes, including immune responses, where they contribute to the elimination of pathogens and play a role in antiviral, antibacterial, and antiparasitic defense mechanisms [5,6]. However, disruption of this redox equilibrium, due to either excessive ROS production by environmental (e.g., smoking, pollution, etc.) or cellular (e.g., inflammation) sources or impaired antioxidant defenses, can result in oxidative stress [4,7] (Figure 1).

In such conditions, elevated ROS levels cause damage to critical biomolecules, including lipids, proteins, and DNA, ultimately compromising cellular integrity and function [8]. Thus, oxidants represent a double-edged sword: while they are indispensable for physiological signaling and host defense at controlled levels, their overproduction can lead to oxidative damage contributing to the pathogenesis of various diseases [9].

## 2. The Oxidative Stress: Cellular Consequences

The cellular consequences of oxidative stress include lipid peroxidation, protein alteration and DNA damage.

**Figure 2:**
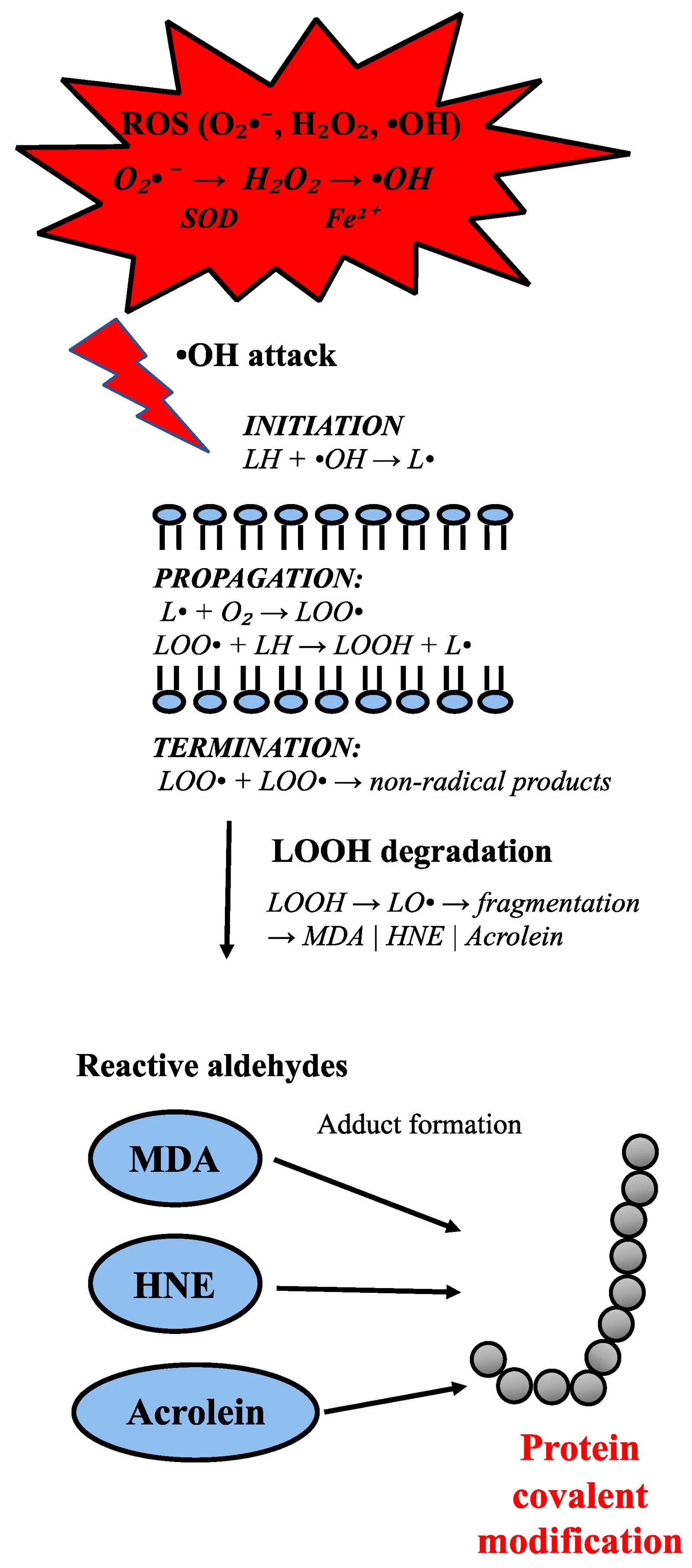
Mechanistic representation of lipid peroxidation induced by ROS (O_2_•^−^, H_2_O_2_, •OH) and formation of secondary reactive aldehydes. Superoxide (O_2_•^−^) is enzymatically dismutated to H_2_O_2_ by superoxide dismutase (SOD), which in the presence of redox-active metals (e.g., Fe^2+^) can undergo Fenton-type reactions to yield the highly reactive hydroxyl radical (•OH). This radical initiates lipid peroxidation by abstracting a hydrogen atom from polyunsaturated fatty acids in membrane lipids (LH), forming lipid radicals (L•) (initiation phase). During the propagation phase, lipid radicals rapidly react with molecular oxygen (O_2_) to form lipid peroxyl radicals (LOO•), which in turn react with adjacent lipids to produce lipid hydroperoxides (LOOH) sustaining the chain reaction. The process is terminated when radical species combine to form non-radical, stable products (termination phase), although this does not prevent the accumulation of lipid hydroperoxides. LOOH can undergo further decomposition into alkoxyl radicals (LO•), which undergo fragmentation to generate reactive aldehydes such as malondialdehyde (MDA), 4-hydroxynonenal (HNE), and acrolein. These electrophilic species readily form covalent adducts with proteins, contributing to structural and functional alterations, enzyme inactivation, and cellular dysfunction.

Lipid peroxidation, a primary form of oxidative damage to cell membranes is a process that begins when ROS attack the double bonds in unsaturated fatty acids within cell membranes [10]. Specifically, the highly reactive hydroxyl radical (•OH) initiates lipid peroxidation by abstracting a hydrogen atom from polyunsaturated fatty acids in membrane lipids (LH) and forming lipid radicals (L•) (initiation phase). Then, lipid radicals rapidly react with molecular oxygen (O_2_) to form lipid peroxyl radicals (LOO•), which in turn react with adjacent lipids to produce lipid hydroperoxides (LOOH) sustaining the chain reaction (propagation phase). The process is terminated when radical species combine to form non-radical, stable products (termination phase), although this does not prevent the accumulation of lipid hydroperoxides. LOOH can further decompose into lipid alkoxyl radicals (LO•), which subsequently fragment to produce reactive aldehydes, including malondialdehyde (MDA), 4-hydroxynonenal (HNE), and acrolein that can readily react with proteins to form covalent adducts, (Figure 2).

These electrophilic molecules are highly reactive and toxic compounds, capable of interfering with various cellular processes leading to changes in protein structure and function, loss of enzymatic activity, and impaired cellular function [11]. In particular, they can covalently modify signaling proteins through post-translational mechanisms, including interactions with nucleophilic amino acid residues such as cysteine, lysine, and histidine. This process, named lipoxidation, can disrupt normal protein function by altering protein structure, stability, or interactions with other molecules [12,13] and influencing several cellular signaling pathways [14,15]. The impaired protein function due to LDA-mediated modifications can contribute to the development of various diseases including cardiovascular disease (CVD) [16], cancer [17], and neurodegenerative disorders [18,19]. Additionally, they can play a role in the onset of conditions like diabetes and diabetic ulcers [20]. Furthermore, these compounds represent potential biomarkers for conditions like Alzheimer’s disease (AD), Parkinson’s disease (PD), Amyotrophic Lateral Sclerosis (ALS), and Huntington’s disease (HD) [21].

**Figure 3:**
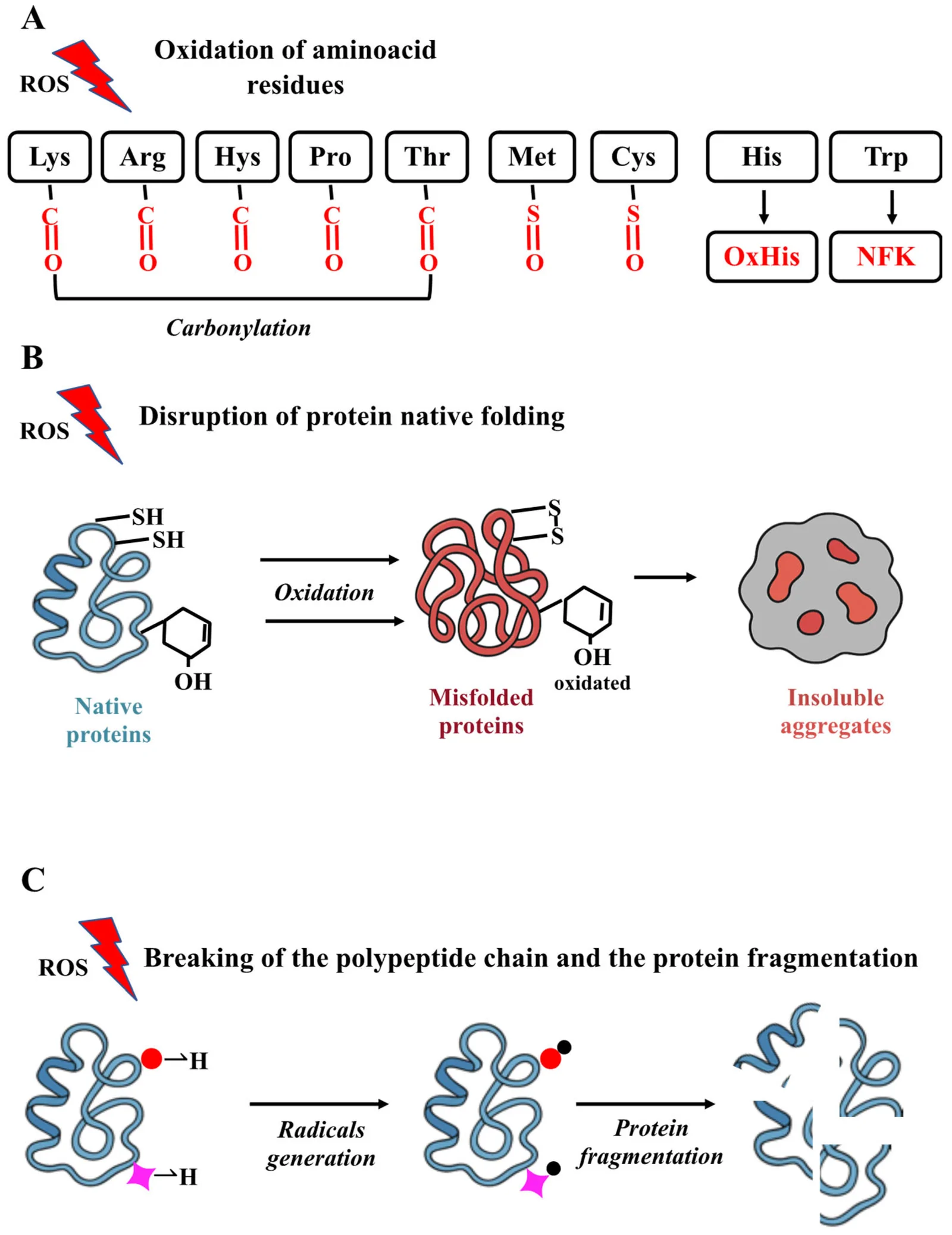
Schematic figure representing protein damage by oxidative stress. ROS damage proteins through oxidation of amino acid side chains (R groups) (**A**), misfolding following oxidation (**B**), radical generation and cleavage of peptide bonds (**C**).

ROS can react with proteins causing oxidation of amino acid residues, misfolding and aggregation between proteins, and cleavage of peptide bonds [22]. Protein carbonylation, a recognized biomarker for oxidative stress, is a type of irreversible oxidative modification that occurs when ROS attack the side chains of lysine, arginine, histidine, proline, and threonine, among several amino acids, forming carbonyl groups, which react with 2,4-dinitrophenylhydrazine (DNPH) to form hydrazones, a method used to detect and quantify these oxidized compounds [22,23]. Additionally, methionine and cysteine undergo oxidation resulting in the formation of methionine sulfoxide and cysteine sulfoxide. Other amino acid residues, such as histidine and tryptophan, can also undergo oxidation, producing 8-oxohistidine (OxHis) and N-formilkynurenina (NFK), respectively, and cysteine side chains may become chlorinated forming chloramine [24] (Figure 3A). Oxidative stress-induced modifications can disrupt protein native folding by destabilizing their three-dimensional structure [25]. Aberrant disulfide bonds form when cysteine thiol groups (-SH) oxidize and link incorrectly, preventing proper protein conformation [26]. Additionally, oxidation of aminoacidic side chains alters their chemical properties, often increasing hydrophobicity or changing polarity. Normally buried within the protein core, these hydrophobic regions become exposed after oxidative damage, promoting abnormal protein–protein interactions. This leads to protein misfolding and the formation of insoluble aggregates that impair cellular function by disrupting proteostasis and overwhelming degradation pathways, ultimately contributing to cellular dysfunction and disease progression (Figure 3B). ROS can also directly attack the peptide backbone by abstracting hydrogen atoms from the α-carbon or amide nitrogen atoms within the polypeptide chain. This generates highly unstable radical species on the protein backbone weakening the peptide bonds and making the peptide bound susceptible to cleavage. The result is the breaking of the polypeptide chain and the protein fragmentation into smaller peptides or even individual amino acids [27] (Figure 3C). These oxidative modifications can have significant effects on protein function. For example, they can alter the conformation of proteins, disrupting their active sites and leading to a loss of enzymatic or structural function [26,28]. Furthermore, oxidative stress can impair protein–protein interactions and signaling pathways, leading to a cascade of cellular dysfunctions [29]. When oxidative damage to proteins becomes extensive, it can cause severe cellular dysfunction and, ultimately, lead to cell death [30,31].

**Figure 4:**
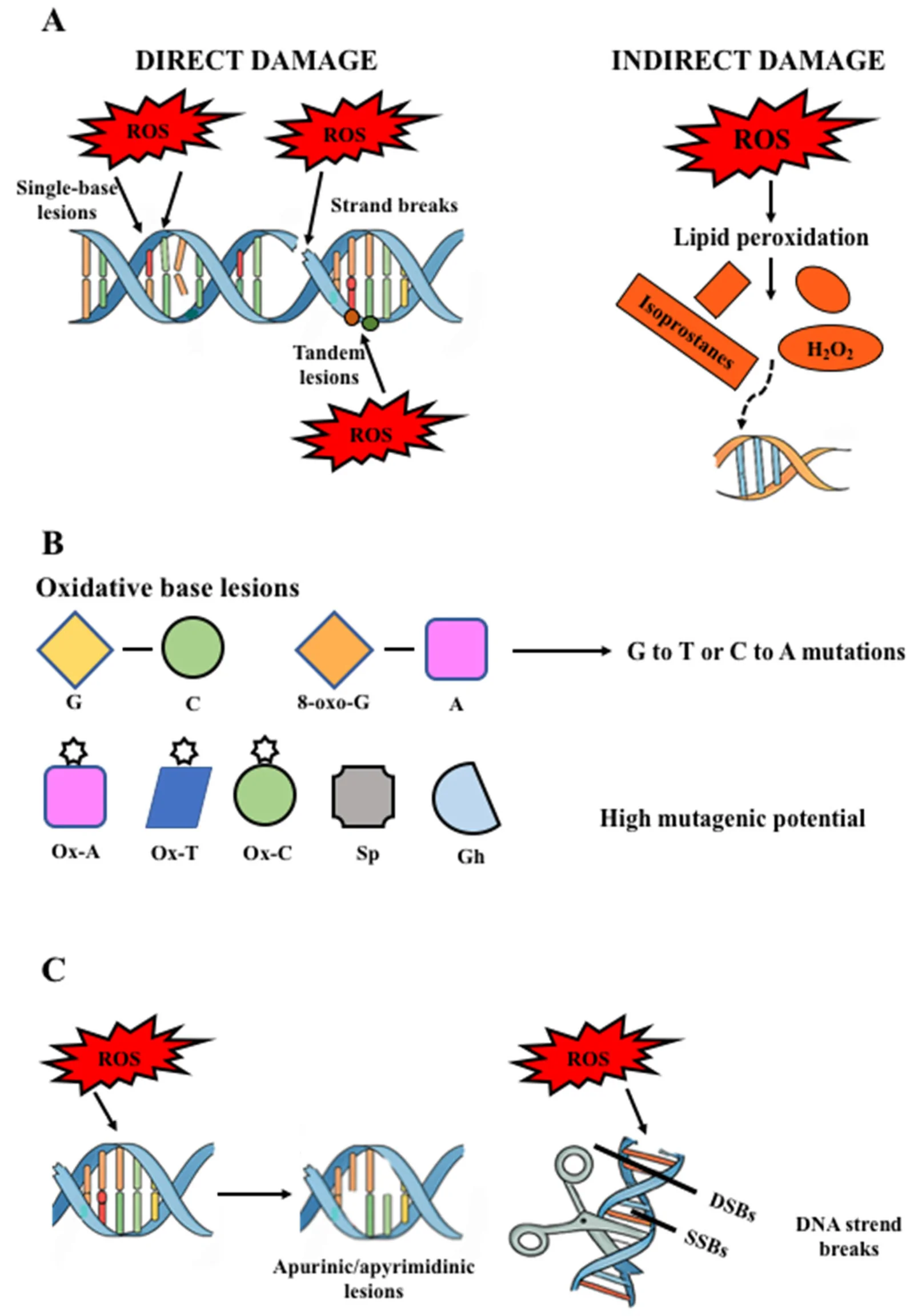
Schematic figure showing DNA damage by oxidative stress. Oxidative stress can induce DNA damage both directly (single-base lesions, tandem lesions, and strand breaks) and indirectly (lesions caused by lipid peroxidation products) (**A**). Species like hydrogen peroxide, can cause oxidative damage and consequent modification of bases, which in turn affects their pairing properties. (**B**). Excess ROS can also induce DNA strand breaks, which can be either single-strand breaks (SSBs) or double-strand breaks (DSBs) (**C**).

Oxidative stress can also induce DNA damage both directly and indirectly. Direct damage includes single-nucleobase lesions, tandem lesions, and strand breaks, while indirect damage results from lipid peroxidation, whose products, such as H_2_O_2_ and isoprostanes can interact with DNA to produce lesions (Figure 4A). However, not all ROS affect DNA in the same way; the specific type of ROS influences the type and location of the resulting DNA damage. Hydroxyl radicals, for instance, can induce strand breaks, base modifications, and abasic sites directly at the site of generation due to their extremely short diffusion range. In contrast, species like hydrogen peroxide, which are more stable and membrane-permeable, can diffuse across cellular compartments and cause oxidative damage more diffusely [32,33]. One of the major consequences of DNA oxidation is the alteration of the chemical structure of the bases, which in turn affects their pairing properties. A key oxidative DNA lesion is the modified base 8-oxoguanine (8-oxo-G), also known as 8-hydroxyguanine or 8-OH-dG, which mispairs with adenine instead of cytosine, leading to G to T or C to A mutations [34] (Figure 4B). In addition to this, more than 20 other oxidative base lesions arise from the attack of ROS on DNA bases have been identified [35]. These lesions include oxidized forms of adenine, thymine, and cytosine and other oxidative base lesions like hydantoin products spiroiminodihydantoin (Sp) and guanidinohydantoin (Gh). These lesions are of interest due to their high mutagenic potential and role in cellular DNA repair pathways [36]. Oxidative stress also leads to the formation of apurinic/apyrimidinic lesions, which occur when the N-glycosidic bond between the nitrogenous base and the deoxyribose sugar is cleaved, leaving behind an intact phosphodiester backbone [37]. Excess ROS can also induce DNA strand breaks, which can be either single-strand breaks (SSBs) or double-strand breaks (DSBs) [38,39] (Figure 4C).

SSBs are more common and generally easier to repair, whereas DSBs are among the most severe forms of DNA damage. To repair oxidative DNA damage, cells rely on the base excision repair/single-strand break repair (BER/SSBR) pathway for repairing oxidized bases, abasic sites, and SSBs [40]. Double-strand breaks are repaired through two primary mechanisms: homologous recombination (HR) and non-homologous end joining (NHEJ). HR is considered to be error-free, whereas NHEJ is considered to be error-prone [41]. The accumulation of unrepaired oxidative DNA lesions can lead to serious biological consequences [42]. Notably, mutations arising from DNA replication errors at damaged sites can alter gene sequences and disrupt gene expression, potentially contributing to the onset and progression of diseases such as cancer. Moreover, oxidative DNA damage has been linked to aging and neurodegenerative disorders, underscoring the significant impact of ROS on human health [43,44,45]. In addition to causing direct DNA damage, oxidative stress can also have a profound impact on the epigenome. ROS are capable of modifying DNA methylation patterns and histone modifications, which in turn can alter gene expression in a heritable, yet reversible, manner. These epigenetic alterations can interfere with normal cellular processes and play a role in the development of various diseases and aging, highlighting the complex ways in which oxidative stress affects both genetic and epigenetic regulation [46].

## 3. Sources of Oxidative Stress

The sources of oxidative stress can be numerous and include both endogenous (internal to the body) and exogenous (external to the body) factors. At the molecular level, ROS are generated by specific cellular systems, including mitochondrial electron transport chain (ETC) leakage, NADPH oxidases (NOX), xanthine oxidase, uncoupled nitric oxide synthase (NOS), and other oxidoreductase enzymes. These sources can be activated or amplified under different physiological and pathological conditions [47].

### 3.1. Endogenous Sources of Oxidative Stress

Even under physiological conditions, oxidative stress and ROS production may increase in response to specific metabolic and cellular stimuli [3]:

• *Metabolic hyperactivity*: conditions associated with increased metabolic demand, such as fever, intense physical exercise, hyperthyroidism, hyperglycemia or excessive caloric intake, can enhance ROS generation, primarily through mitochondrial mechanisms. Elevated substrate availability increases electron flux through the mitochondrial electron transport chain (ETC), promoting electron leakage at complexes I and III and subsequent superoxide production [48]. Hyperglycemia contributes to this process by further stimulating mitochondrial ROS production particularly pronunced in pancreatic β-cells. Moreover, chronic overnutrition or high-fat diets may impair mitochondrial function, leading to reduced ATP synthesis and additional ROS production [49,50,51].
• *Inflammatory processes*: inflammation is a protective response of the organism to a wide range of factors, such as infections, allergens, radiations, chronic diseases, obesity, alcohol, tobacco, aimed at removing pathogens, repairing damaged tissues, and protecting the organism. During this process, activated immune cells such as neutrophils and macrophages produce ROS via NADPH oxidases (particularly NOX2) as part of the antimicrobial defense response [52]. While acute inflammation is typically self-limiting, persistent activation leads to chronic inflammation, characterized by sustained ROS production. This prolonged oxidative environment can damage surrounding tissues and contribute to systemic oxidative stress, thereby promoting the development of chronic diseases such as rheumatoid arthritis, type 2 diabetes (T2D), and ulcerative colitis [53].

### 3.2. Exogenous Sources of Oxidative Stress

Various external factors, referred to as exogenous sources, can significantly enhance ROS generation by directly producing reactive species or by activating endogenous ROS-generating systems [54]. Environmental stressors that contribute to increased ROS production include ultraviolet (UV) radiation, ionizing radiation (IR), pollutants, and lifestyle. In addition, xenobiotics such as drugs, food additives and certain pharmaceutical treatments (i.e., antiblastic drugs) can contribute to oxidative stress [55].

• *Ultraviolet (UV) radiation exposure:* UV radiation, particularly UVA and UVB, induces the generation of ROS in exposed tissues, especially in the skin. These effects are mediated both by direct photochemical reactions and by activation of endogenous chromophores such as riboflavin and porphyrins, as well as enzymes including NADPH oxidases. UV exposure promotes lipid peroxidation, DNA damage (e.g., thymine dimers and 8-oxo-guanine formation), and depletion of intracellular antioxidants such as glutathione (GSH) [56,57]. These processes contribute to photoaging and increase the risk of skin cancers, including basal cell carcinoma, squamous cell carcinoma, and melanoma [58].
• *Ionizing Radiation (IR):* Ionizing radiation is also recognized as an environmental stressor that can significantly increase the production of ROS, acting both through direct ionization of biomolecules and indirectly via radiolysis of water, leading to the formation of hydroxyl radicals, superoxide anions, and hydrogen peroxide [59]. These reactive species can further interact with transition metals such as iron and copper, amplifying oxidative damage through Fenton-type reactions. Experimental studies have shown increased intracellular ROS levels and peroxide production following radiation exposure which has also been linked to tissue damage [60,61], and a correlation between IR induced oxidative stress and cardiotoxicity [60,61,62].
• *Environmental pollution:* environmental pollutants represent important exogenous source of ROS. They include fine particulate matter, heavy metals (e.g., lead, mercury, cadmium), volatile organic compounds (e.g., benzene, formaldehyde) and chemical compounds from cigarette smoke [63,64,65,66]. These agents can directly generate ROS or activate cellular sources such as NADPH oxidases and mitochondrial pathways. For instance, inhaled particulate matter and ozone can penetrate the respiratory tract and enter systemic circulation, directly generating ROS and activating pulmonary inflammatory responses, thereby contributing to chronic respiratory diseases, cardiovascular disorders, and increased cancer risk, particularly lung cancer [67,68].
• *Lifestyle:* lifestyle factors critically influence oxidative stress by modulating the balance between ROS production and antioxidant defenses. Unfavorable habits such as smoking, poor diet, physical inactivity, and exposure to environmental pollutants can induce the generation of ROS and pro-oxidant compounds, including reactive chemicals and heavy metals, which impair antioxidant systems and promote chronic inflammation and tissue damage. In contrast, a healthy lifestyle including balanced nutrition, regular physical activity, and avoidance of tobacco and environmental toxins, supports the body’s antioxidant systems and helps maintain oxidative balance [55].

*Cigarette smoke* induces oxidative stress by delivering thousands of compounds, including ROS, heavy metals, tar, nicotine, and polycyclic aromatic hydrocarbons. In addition to directly introducing reactive species, cigarette smoke can activate endogenous ROS-generating systems, such as NADPH oxidases and mitochondrial pathways. This results in impairing antioxidant defenses, promotion of chronic airway inflammation, and damage to lung epithelial cells. Furthermore, oxidative mechanisms induced by smoking contribute to collagen degradation and premature skin aging, and are strongly associated with an increased risk of cancers, particularly in the lungs, skin, oral cavity, and bladder [69].

*Unbalanced Diet* high in saturated and trans fats (e.g., red meat, fried and processed foods) and in processed sugars, enhance systemic inflammation and ROS production by increasing mitochondrial electron flux and promoting electron leakage from the electron transport chain, as well as by activating inflammatory pathways and NADPH oxidases. Conversely, a diet low in antioxidant-rich foods (e.g., fruits, vegetables, whole grains) reduces intake of essential vitamins, polyphenols, and minerals required for oxidative defense [70]. Overweight and obesity represent chronic inflammatory conditions in which adipose tissue releases pro-inflammatory cytokines, such as TNF-α and IL-6, thereby enhancing ROS production through both mitochondrial dysfunction and enzymatic sources and promoting oxidative stress. This oxidative environment is further exacerbated by obesity-associated insulin-resistance, which contributes to the onset and progression of metabolic and CVD, including hypertension, dyslipidemia, T2D, atherosclerosis, and nonalcoholic fatty liver disease [71].

*Sedentary behavior* is recognized as a contributor to oxidative stress, as it is associated with impaired mitochondrial function and disruption of cellular redox balance, similarly to other detrimental lifestyle factors. Decreased mitochondrial activity can lead to increased electron leakage and ROS generation, along with disruption of cellular redox balance. In contrast, regular physical activity enhances mitochondrial biogenesis and improves the coordination between ROS production and antioxidant defenses, particularly in skeletal muscle [72,73]. Sedentary behavior is also associated with chronic low-grade inflammation, further amplifying ROS production and increasing susceptibility to oxidative damage.

## 4. Oxidative Stress in Aging

Oxidative stress is a key factor in aging, a continuous and progressive process which involves a gradual decline in physiological function and contributes to the development and advancement of various age-related diseases [74]. It is characterized by numerous molecular alterations within cells, collectively referred to as the hallmarks of aging. Several hallmarks have been identified, including genomic instability, telomere shortening, epigenetic alterations, loss of proteostasis, deregulation of nutrient sensing, mitochondrial dysfunction, cellular senescence, stem cell exhaustion, altered intercellular communication, and chronic inflammation. They are closely interlinked, influencing one another in complex ways and contributing to the aging process as a whole [75,76,77]. A central feature of aging is the progressive accumulation of ROS, which contributes to multiple hallmarks through distinct but interconnected mechanisms. ROS can directly damage telomeric DNA, which is particularly susceptible to oxidative lesions, thereby accelerating telomere shortening and promoting replicative senescence. At the mitochondrial level, oxidative damage to mitochondrial DNA (mtDNA), proteins, and lipids impairs electron transport chain efficiency, leading to further ROS production and establishing a self-amplifying cycle of mitochondrial dysfunction [77]. This mitochondrial impairment contributes not only to further oxidative stress but also to the development of cellular senescence, thereby linking mitochondrial dysfunction to telomere instability and loss of proliferative capacity. Experimental evidence has shown that mitochondrial dysfunction and increased ROS production contribute to telomere dysfunction and cellular senescence, and that mitochondria-derived ROS can induce preferential telomeric damage under controlled experimental conditions [77].

Elevated ROS levels promote the induction of cellular senescence and the development of the senescence-associated secretory phenotype (SASP), characterized by the release of pro-inflammatory cytokines, chemokines, and proteases that contribute to tissue dysfunction and chronic inflammation. Oxidative stress also influences cellular metabolism through the depletion of nicotinamide adenine dinucleotide (NAD^+^), partly via the activation of NAD^+^-consuming enzymes such as poly(ADP-ribose) polymerases (PARPs) in response to DNA damage. Reduced NAD^+^ availability negatively affects sirtuin activity, thereby impairing mitochondrial homeostasis and stress resistance pathways.

Collectively, these mechanisms highlight the central role of oxidative stress in driving the functional decline associated with aging and in modulating the complex interplay among its major hallmarks. Changes in oxidative stress biomarkers have been consistently observed with advancing age in humans, and alterations in stress response capacity further support the link between oxidative imbalance and biological aging [78,79,80]. Moreover, age-related conditions such as cardiac ischemia and stroke are closely associated with oxidative stress-mediated damage.

## 5. Oxidative Stress in Cardiovascular Diseases (CVDs)

Oxidative stress plays a central role in the biological processes underlying many chronic diseases, significantly contributing to both their initiation and progression. Prolonged exposure to oxidative stress can trigger chronic inflammation, a key driver behind a wide range of chronic conditions including CVD [52]. Conditions that serve as precursors or risk factors for CVD, such as hypertension, atherosclerosis, and diabetes mellitus, are commonly associated with increased oxidative stress [63,81] through its influence on elevated blood pressure and the buildup of arterial plaques [82,83]. Moreover, oxidative stress can alter gene expression and promote the release of inflammatory molecules like cytokines, linking ROS to the activation, dysfunction, and inflammation of the vascular endothelium [84]. High blood glucose levels induce oxidative stress, which is a major contributor to the cardiovascular complications seen in diabetic patients [85]. ROS also play a causal role in various forms of insulin resistance, connecting oxidative stress to both the progression of T2D and hyperglycemic states [86].

## 6. Oxidative Stress in Neurodegenerative Disorders (NDs)

There is a strong connection between oxidative stress and neurodegenerative disorders (NDs), including Parkinson’s disease, Alzheimer’s disease, amyotrophic lateral sclerosis (ALS), multiple sclerosis, and depression [87,88,89]. In Alzheimer’s disease, oxidative damage is a crucial factor driving neuronal death and the worsening of dementia, with the toxic amyloid-beta peptide being generated as a consequence of free radical activity [90,91]. Parkinson’s disease involves oxidative stress in the degeneration of both dopaminergic and non-dopaminergic neurons, with abnormal activation of NADPH oxidase in microglial cells playing a role in the neurodegenerative process targeting dopaminergic neurons [92]. Oxidative stress is also linked to the inflammatory and neurodegenerative changes seen in multiple sclerosis [93] and depression [94]. It contributes to neuroinflammatory responses after events such as cerebral ischemia by affecting the activation of astrocytes and microglia, which leads to increased production of inflammatory mediators [95].

## 7. Oxidative Stress in Chronic Inflammatory Diseases

Chronic respiratory diseases like asthma and chronic obstructive pulmonary disease (COPD), both characterized by ongoing systemic and localized inflammation, are also associated with oxidative stress [96,97]. ROS can hinder the resolution of inflammation by impairing the phagocytic function of alveolar macrophages, which contributes to tissue necrosis and the development of emphysema. Similarly, prolonged exposure to cigarette smoke disrupts the phagocytic and antigen-presenting activities of polymorphonuclear neutrophils (PMNs) and alveolar macrophages, potentially increasing susceptibility to bacterial and viral infections [97]. Free radicals are implicated in the onset and progression of rheumatoid arthritis (RA) by amplifying inflammation, damaging joint tissue, stimulating autoimmunity, and promoting bone destruction. This makes oxidative stress both a consequence and a driver of RA progression [98]. Additionally, oxidative stress is a key contributor to the progression of chronic kidney disease (CKD), directly through damage to the glomeruli and reduced blood flow to the kidneys, and indirectly via its association with inflammation, high blood pressure, and endothelial impairment. Patients with CKD often experience persistent inflammation, marked by the activation of PMNs and monocytes. These immune cells elevate the production of Nicotinamide adenine dinucleotide phosphate (NADPH) oxidase and myeloperoxidase (MPO), enzymes that promote the generation of ROS. In addition, leukocytes from CKD patients release superoxide anions, which neutralize nitric oxide (NO), impairing vasodilation and thereby contributing to the development of hypertension [99].

## 8. Oxidative Stress in Cancer

Oxidative stress is associated with various types of cancer, including prostate, ovarian, liver, lung, and colorectal cancers [100,101,102]. It plays a complex role in tumor development, progression, metastasis and drug resistance through multiple mechanisms [103,104]. As above described, ROS can cause DNA damage, such as mutations, strand breaks, and chromosomal aberrations, thereby compromising genomic integrity [38,39]. These alterations can trigger the activation of oncogenes and the inactivation of tumor suppressor genes, creating conditions that promote uncontrolled cell proliferation and tumor development [105,106]. High ROS levels also affect signaling pathways involved in proliferation, survival, and apoptosis, including Kelch-like ECH-associated protein 1/nuclear factor erythroid 2-related factor 2 (Keap1-NRF2) [107], p53 [108], Adenomatous Polyposis Coli (APC) [109], p21 [110], phosphoinositide-3-kinase (PI3K)/AKT, mitogen activated-protein kinase/extracellular-regulated kinase 1/2 (MAPK/ERK1/2) and protein kinase D (PKD) [100]. These processes can lead to dysregulated cell growth and evasion of programmed cell death. A growing body of evidence supports the existence of a crosstalk between chronic inflammation and cancer [111,112]. Oxidative stress is a key driver of this process, as it promotes the activation of pro-inflammatory transcription factors, thereby enhancing the expression of inflammatory mediators and fostering the establishment of a tumor-promoting microenvironment. Inflammation not only predisposes individuals to malignant transformation but also facilitates multiple stages of tumorigenesis, including cell proliferation, survival, invasion, and metastatic dissemination [113,114,115].

Oxidative stress contributes to cancer development also through the stimulation of tumor angiogenesis and the impairment of the immune system [116]. Tumor cells secrete endothelial growth factors that drive angiogenesis, a process essential for tumor growth [117]. In prostate carcinoma, vascular endothelial growth factor-A (VEGF-A) modulates the Rac1 signaling pathway via vascular endothelial growth factor receptor 2 (VEGFR2), inducing NOX2-mediated ROS [118]. Other cytokines, including fibroblast growth factor (FGF) and hepatocyte growth factor (HGF), similarly promote angiogenic signaling through ROS induction, and ROS can further potentiate growth factor receptor signaling, thereby enhancing tumor progression [118]. Under hypoxic conditions, ROS cooperate with hypoxia-inducible factor-1 (HIF-1) to augment VEGF activity, amplifying angiogenesis and facilitating metastatic spread [119].

Beyond their role in neovascularization, excessive levels of ROS can compromise the function of immune cells, such as T cells, natural killer (NK) cells, and macrophages, leading to immune suppression and promoting tumor cell growth and survival. ROS, particularly mitochondrial ROS (mtROS), inflict oxidative damage on mitochondrial DNA (mtDNA) and activate interferon (IFN) signaling, leading to PD-L1 upregulation and suppression of T cell activation, dendritic cell (DC) function, and M1 macrophage activity [120]. In tumors, ROS-activated mitochondrial Lon protease triggers inflammatory cytokines release via the mtROS–NF-κB pathway, promoting immunosuppression in macrophages and DCs and driving the differentiation of T cells into regulatory T cells (Tregs) [121,122,123]. NF-κB directly regulates Lon transcription, creating a positive feedback loop that amplifies mtROS production [124]. Elevated mtROS promotes the cytosolic release of oxidized mtDNA, which activates the cGAS–STING–TBK1 axis, inducing type I interferons (IFNs) that further increase PD-L1 and IDO-1 expression, suppressing the antitumor function of CD8+ T cells. mtROS accumulation also stimulates the secretion of extracellular vesicles (EVs) containing oxidized mtDNA and PD-L1; once internalized by tumor-associated macrophages (TAMs), EVs induce of IFN-γ and IL-6 release promoting macrophage polarization toward the M (IL-4) phenotype [124]. Hypoxia, via HIF-1α, further enhances Lon expression and IL-6 secretion through STAT3, while ROS- and hypoxia-driven Lon upregulation increase the secretion of EVs carrying mtDNA and PD-L1 [125]. Together, the angiogenic and immunosuppressive pathways converge, creating a self-reinforcing network that sustains tumor vascularization, suppresses antitumor immunity, and accelerates malignant progression.

Although, as described above, ROS can initiate and promote tumorigenesis, high levels of ROS can be cytotoxic to tumor cells and exert inhibitory effects at various stages of cancer [101,126]. Indeed, ROS can stimulate apoptosis in tumor cells by activating multiple metabolic pathways, as well as the tumor suppressor protein p53, which in turn promotes ROS production [127,128]. ROS can also trigger ferroptosis, an iron-dependent form of cell death distinct from apoptosis, involving the redox cycling of Fe^2+^/Fe^3+^ and the peroxidation of membrane phospholipids [129,130]. Oxidative stress can also act as a barrier to metastatic dissemination. Piskounova and colleagues demonstrated in a murine melanoma model that high ROS levels limit the metastatic spread of melanoma cells and that treatment with N-acetylcysteine (NAC) increases circulating melanoma frequency and metastatic disease burden [131]. These observations highlight the potential antitumor role of oxidative stress at specific stages of cancer progression.

## 9. Antioxidants: Classifications, Mechanisms, and Therapeutic Implications

Antioxidants constitute a heterogeneous group of compounds which can delay or prevent the oxidation of a substrate and counteract oxidative stress by neutralizing oxidants such as ROS [132,133]. Antioxidants can act at several different levels in the oxidative sequence by using multiple mechanisms of action. They differ in structure and origin and are classified into endogenous and exogenous antioxidants.

### 9.1. Endogenous Antioxidants

Endogenous antioxidants are synthesized within the body and comprise both enzymatic and non-enzymatic components [134,135]. The enzymatic antioxidants include SODs, catalase, and glutathione peroxidase (GPx). These enzymes catalyze the conversion of reactive intermediates into less harmful species. For instance, they transform superoxide anion (O_2_^−^) into hydrogen peroxide (H_2_O_2_), which is then converted into water through a multi-step process involving cofactors such as iron, zinc, copper, and manganese [1]. Non-enzymatic antioxidants include GSH, ubiquinone, uric acid, bilirubin, and albumin, which directly scavenge free radicals and ROS neutralizing them and preventing chain reactions. Moreover, they can donate hydrogen atoms to stabilize reactive radicals blocking their propagation [2].

### 9.2. Exogenous Antioxidants

Exogenous antioxidants are obtained from dietary sources such as fruits and vegetables, and plant-derived products [136]. They primarily act as free radical scavengers, neutralizing harmful molecules and preventing them from damaging cells and tissues. They can also enhance the effectiveness of endogenous antioxidant systems by working synergistically with the body’s own antioxidant systems [134]. Exogenous antioxidants include:

• *Vitamins:* vitamin C and vitamin E, the latter being a lipid-soluble antioxidant that serves as a primary defense against lipid peroxidation. They also exhibit anti-inflammatory activity by reducing monocyte recruitment and scavenger receptor expression on macrophages [137,138].
• *Polyphenols and phytochemicals:* these include flavonoids (e.g., quercetin in onions, apples, berries), catechins (in green tea), anthocyanins (in berries, red cabbage), phenolic acids (e.g., caffeic and ferulic acid), tannins (in tea and wine), resveratrol (in grapes, red wine), and curcumin (in turmeric) [139,140].
• *Carotenoids*: they are pigments responsible for the vibrant colors in many fruits and vegetables, and their antioxidant properties contribute to various health benefits. They include β-carotene, lycopene, lutein, zeaxanthin [141].

Antioxidants exert protective effects through several mechanisms including [142]:

• *Direct scavenging of free radicals:* antioxidants can effectively stop the chain reactions that lead to cellular damage by donating an electron to a free radical, stabilizing it and rendering it harmless. This reaction can also create a less reactive radical, which may be further neutralized by other antioxidants or through other cellular mechanisms.
• *Chelation of metal ions:* antioxidants bind to metal ions (like iron and copper), and prevent these metals from catalyzing the production of harmful free radicals through reactions like the Fenton reaction.
• *Delay in formation of secondary reactive species:* antioxidants can interfere with the reactions that create secondary reactive species, preventing the propagation of oxidative damage.
• *Interruption of chain propagation reactions (chain-breaking antioxidants):* chain-breaking antioxidants act as radical scavengers, terminating free radical chain reactions before they can damage other molecules.
• *Upregulation of endogenous antioxidant defenses:* Antioxidants can stimulate the production and activity of the body’s own antioxidant enzymes, such as SOD, catalase, and GPx.

Some antioxidants exhibit a dual role in redox biology, acting as protective agents against oxidative stress under physiological conditions while potentially displaying pro-oxidant behavior under specific circumstances [143]. This paradoxical activity is influenced by factors such as antioxidant concentration, the presence of transition metal cations (e.g., iron and copper), lipid hydroperoxides, and local oxygen levels [143]. At elevated concentrations or in the presence of redox-active metals, antioxidants like vitamin C, flavonoids, and tocopherols can undergo redox cycling, donating electrons that generate ROS such as hydroxyl radicals, superoxide, and hydrogen peroxide [144,145,146].

Notably, certain polyphenols and flavonoids exhibit biphasic effects, acting as mild pro-oxidants at low concentrations while triggering endogenous antioxidant defenses at higher doses [145,147]. This dual functionality has significant implications for health, as excessive ROS production is linked to pathologies such as CVD, neurodegeneration, and cancer, as described above. Thus, a nuanced understanding of the context-dependent redox behavior of antioxidants is essential for their effective therapeutic application and for guiding future research into their role in disease prevention and management.

## 10. Nutrition and Oxidative Stress

Oxidative stress is strongly influenced by nutrition. A diet rich in antioxidants, such as vitamins C and E, polyphenols, and carotenoids, predominantly found in fruits and vegetables, helps neutralize harmful free radicals and supports the body’s antioxidant defense systems. Recent studies have shown that plant-based dietary patterns are associated with lower levels of oxidative stress and pro-inflammatory biomarkers [148]. In particular, the Mediterranean diet and the DASH diet (Dietary Approaches to Stop Hypertension) have been inversely associated with these biomarkers in both observational and interventional studies [149]. The Mediterranean diet, characterized by a high intake of fruits, vegetables, whole grains, legumes, nuts, and seeds, with olive oil as the primary fat source, has been consistently linked to reduced risk of chronic diseases and lower all-cause mortality [148]. Its health benefits are largely attributed to its high antioxidant capacity, reflected in reduced lipid peroxidation markers, such as F2-isoprostanes, ox-LDL (low-density lipoproteins), and MDA [150] and oxidative DNA damage (e.g., 8-OHdG) [151], along with increased endogenous antioxidant defenses, including total antioxidant capacity (TAC), SOD, and catalase activity [152]. Similarly, the DASH diet, rich in fruits and vegetables and low in sodium, has demonstrated efficacy in reducing oxidative stress and inflammation by lowering lipid peroxidation biomarkers and enhancing antioxidant status (e.g., glutathione-GSH; and nitric oxide levels) [153,154]. Key components of these beneficial diets include the polyphenols, fibers, and Omega-3 fatty acids. Polyphenols, widely present in fruits, vegetables, tea, wine, and cocoa, function as direct antioxidants and also modulate gene expression related to antioxidant defense [155,156]. Dietary fibers support gut health and promote the production of short-chain fatty acids (SCFAs), which exert both antioxidant and anti-inflammatory effects, in part by facilitating the release of antioxidants through microbial fermentation [157]. Omega-3 fatty acids, found in fish, some nuts, and vegetable oils, are known for their anti-inflammatory properties and their ability to reduce oxidative stress [158]. In contrast, Western-style and fast-food diets, characterized by high intake of saturated fats, refined sugars, and animal proteins, and low in dietary fiber, are associated with increased oxidative stress and inflammation. These dietary patterns are linked to elevated lipid peroxidation and inflammatory markers and decreased antioxidant defenses [159]. Highly processed foods, which are devoid of fiber and antioxidants, contribute to a pro-oxidative environment [160]. Excessive intake of refined carbohydrates and alcohol can also exacerbate oxidative stress and cellular damage [159,161].

Weight loss, whether through hypocaloric diets or physical activity, plays a significant role in improving redox status and reducing oxidative stress markers. It can improve redox status by decreasing free radical production, increasing antioxidant defenses, and reducing inflammation. This is particularly important in individuals with obesity. In fact, obesity is linked to increased oxidative stress, and weight reduction strategies can help restore the balance between oxidants and antioxidants, ultimately improving overall health [162].

Postprandial oxidative stress is a distinct form of nutritional oxidative stress, characterized by the increased production of ROS and other free radicals. It results from a redox imbalance, which may be triggered by an excessive oxidative burden following the consumption of meals rich in fats and sugars, or by an inadequate intake of nutrients that counteract pro-oxidant processes [163]. Macronutrients influence redox homeostasis in the body, either by becoming targets of oxidative modifications after absorption or by being present in the diet in a pro-oxidant form [164]. Postprandial oxidative stress is particularly relevant in Western societies, where the habit of consuming frequent meals rich in refined carbohydrates leads to prolonged postprandial periods, glycemic spikes, and consequently increased oxidative stress [165]. A sustained postprandial hyperlipidemia and/or hyperglycemia are strongly associated with a higher risk of atherosclerosis, T2D, and obesity [166,167,168]. LDL are a primary target of postprandial oxidative modification. The ingestion of oxidized or oxidizable dietary lipids, including unsaturated fatty acids incorporated into LDL, contributes to the pro-oxidant load and the formation of oxidized LDL, a well-established atherogenic factor [169]. Moreover, postprandial hyperglycemia, or high blood sugar after eating, is a strong predictor of cardiovascular risk and can exert a direct toxic effect on the vascular endothelium through mechanisms mediated by oxidative stress, independently of other risk factors [170]. It is observed that in individuals experiencing hyperlipidemia and hyperglycemia, endothelium-dependent vasodilation becomes compromised in the postprandial phase, thereby establishing postprandial oxidative stress as a pivotal determinant in influencing cardiovascular risk [171,172]. In addition, a single high-fat meal can transiently impair endothelial function even in healthy individuals [173,174].

Postprandial oxidative stress can be attenuated by consuming dietary antioxidants alongside meals high in oxidized or oxidizable fats. Flavonoids, a key group of these compounds, are known for their antioxidant and anti-inflammatory effects, which may contribute to cardiovascular protection. Research indicates that polyphenols, such as those found in wine, cocoa, or tea, can support endothelial function, decrease the oxidation of LDL cholesterol, and promote better cardiovascular outcomes [175,176]. For instance, a moderate intake of red wine (e.g., 300 mL during meal) has been demonstrated to mitigate the postprandial increase in LDL oxidation susceptibility and to protect diabetic patients from meal-induced oxidative stress [175,176,177]. Similarly, the consumption of flavanol-rich cocoa or tea has been associated with a reversal of endothelial dysfunction and a reduction in LDL oxidation [176,178].

A varied intake of antioxidant compounds is more effective at counteracting the oxidative stress caused by fats and sugars after meals than relying on a single antioxidant, largely due to the diversity of human diets. Since different foods contain varying types and amounts of fats and sugars, the resulting oxidative stress can differ in both intensity and nature. Because antioxidants act through different mechanisms and have unique abilities to neutralize free radicals, combining multiple antioxidants provides more comprehensive protection against a broader range of harmful molecules. Consuming a diverse diet rich in various antioxidants ensures the body is better equipped to manage the wide array of free radicals produced by different foods [171].

## 11. Conclusions

This review provides an integrated and updated overview of oxidative stress across multiple biological levels, linking molecular mechanisms of ROS generation and detoxification with their systemic implications in aging and major chronic diseases. In particular, it emphasizes the dual role of ROS as both signaling molecules and mediators of cellular damage, and highlights the complex interplay between mitochondrial dysfunction, inflammation, and metabolic dysregulation. Particular attention should also be given to the context-dependent dual role of some redox-active molecules, which may display both antioxidant and pro-oxidant properties, with potential translational implications in biomedical, pharmacological, and environmental applications. A key contribution of this review is the incorporation of emerging concepts such as redox-dependent epigenetic regulation, postprandial oxidative stress, and the context-dependent dual behavior of antioxidants. Key takeaways include: (i) the central role of mitochondrial ROS in driving both physiological signaling and pathological processes; (ii) the importance of lipid peroxidation-derived products as active mediators of cellular dysfunction rather than passive byproducts; (iii) the contribution of lifestyle factors, particularly diet and metabolic status, in modulating redox homeostasis; and (iv) the need to consider oxidative stress as a dynamic and context-dependent process rather than a purely detrimental condition. Despite significant progress, several gaps remain in the current literature. These include a limited understanding of the precise thresholds distinguishing physiological from pathological ROS levels, incomplete characterization of tissue-specific redox responses, and insufficient clinical translation of antioxidant-based interventions, many of which have shown inconsistent or inconclusive results. Furthermore, the dual antioxidant/pro-oxidant nature of many compounds remains poorly defined in vivo, highlighting the need for more targeted and personalized approaches. Future research should focus on identifying reliable biomarkers of oxidative stress, clarifying causal relationships in human disease, and developing mechanism-based therapeutic strategies that move beyond non-specific antioxidant supplementation.

## Figures and Tables

**Table 1:** Pro-oxidant and Antioxidant systems. The table summarizes the main components of the cellular redox system, distinguishing between pro-oxidant species and antioxidant defenses.

Pro-Oxidant System	Antioxidant	
Oxygen-derived radicals	Enzymatic components	Non-enzymatic components
superoxide (O_2_^−^)	Catalase	Glutathione
hydroxyl radical (•OH)	superoxide dismutase (SOD)	vitamin C (ascorbic acid)
hydrogen peroxide (H_2_O_2_)	glutathione peroxidase	vitamin E (tocopherol)

## Data Availability

No new data were created or analyzed in this study. Data sharing is not applicable to this article.

## Abbreviations

The following abbreviations are used in this manuscript:

8-oxo-G	8-oxoguanine
AD	Alzheimer’s disease
ALS	Amyotrophic Lateral Sclerosis
APC	Adenomatous Polyposis Coli
COPD	chronic obstructive pulmonary disease
Cu	copper
CKD	chronic kidney disease
CVD	cardiovascular disease
DASH	Dietary Approaches to Stop Hypertension
DC	dendritic cell
DNPH	2,4-dinitrophenylhydrazine
DSBs	double-strand breaks
EVs	extracellular vesicles
Fe	iron
FGF	fibroblast growth factor
Gh	guanidinohydantoin
GPx	glutathione peroxidase
GSH	glutathione
H_2_O_2_	hydrogen peroxide
HD	Huntington’s disease
HGF	hepatocyte growth factor
HIF-1	hypoxia-inducible factor-1
HNE	4-hydroxynonenal
HR	homologous recombination
Keap1-NRF2	Kelch-like ECH-associated protein 1/nuclear factor erythroid 2-related factor 2
IFN	interferon
IR	ionizing radiation
LDA	lipid aldehydes
LDL	low-density lipoproteins
MAPK/ERK1/2	mitogen activated-protein kinase/extracellular-regulated kinase 1/2
MDA	malondialdehyde
MPO	myeloperoxidase
mtDNA	mitochondrial DNA
mtROS	mitochondrial ROS
NAC	N-acetylcysteine
NADPH	Nicotinamide adenine dinucleotide phosphate
NFK	N-formilkynurenina
NHEJ	non-homologous end joining
NK	natural killer
NO	nitric oxide
O_2_^−^	superoxide anion
OxHis	8-oxohistidine
PD	Parkinson’s disease
PI3K	phosphoinositide-3-kinase
PKD	protein kinase D
PMNs	polymorphonuclear neutrophils
RA	rheumatoid arthritis
ROS	Reactive oxygen species
SCFAs	short-chain fatty acids
SOD	superoxide dismutase
Sp	spiroiminodihydantoin
SSBs	single-strand breaks
T2D	type 2 diabetes
TAC	total antioxidant capacity
TAMs	tumor-associated macrophages
Tregs	regulatory T cells
UV	ultraviolet radiation
VEGF-A	vascular endothelial growth factor-A
VEGFR2	vascular endothelial growth factor receptor 2

## References

[1] Ighodaro O.M., Akinloye O.A. (2018). First line defence antioxidants-superoxide dismutase (SOD), catalase (CAT) and glutathione peroxidase (GPX): Their fundamental role in the entire antioxidant defence grid. Alex. J. Med..

[2] Mironczuk-Chodakowska I., Witkowska A.M., Zujko M.E. (2018). Endogenous non-enzymatic antioxidants in the human body. Adv. Med. Sci..

[3] Aramouni K., Assaf R., Shaito A., Fardoun M., Al-Asmakh M., Sahebkar A., Eid A.H. (2023). Biochemical and cellular basis of oxidative stress: Implications for disease onset. J. Cell. Physiol..

[4] Chandimali N., Bak S.G., Park E.H., Lim H.J., Won Y.S., Kim E.K., Park S.I., Lee S.J. (2025). Free radicals and their impact on health and antioxidant defenses: A review. Cell Death Discov..

[5] Herb M., Schramm M. (2021). Functions of ROS in Macrophages and Antimicrobial Immunity. Antioxidants.

[6] Andrés C.M.C., Pérez de la Lastra J.M., Juan C.A., Plou F.J., Pérez-Lebeña E. (2022). The role of reactive species on innate immunity. Vaccines.

[7] Pizzino G., Irrera N., Cucinotta M., Pallio G., Mannino F., Arcoraci V., Squadrito F., Altavilla D., Bitto A. (2017). Oxidative Stress: Harms and Benefits for Human Health. Oxidative Med. Cell. Longev..

[8] Jena A.B., Samal R.R., Bhol N.K., Duttaroy A.K. (2023). Cellular Red-Ox system in health and disease: The latest update. Biomed. Pharmacother..

[9] Wang Y., Qi H., Liu Y., Duan C., Liu X., Xia T., Chen D., Piao H.L., Liu H.X. (2021). The double-edged roles of ROS in cancer prevention and therapy. Theranostics.

[10] Ayala A., Muñoz M.F., Argüelles S. (2014). Lipid peroxidation: Production, metabolism, and signaling mechanisms of malondialdehyde and 4-hydroxy-2-nonenal. Oxidative Med. Cell. Longev..

[11] Moldogazieva N.T., Zavadskiy S.P., Astakhov D.V., Terentiev A.A. (2023). Lipid peroxidation: Reactive carbonyl species, protein/DNA adducts, and signaling switches in oxidative stress and cancer. Biochem. Biophys. Res. Commun..

[12] Gęgotek A., Skrzydlewska E. (2019). Biological effect of protein modifications by lipid peroxidation products. Chem. Phys. Lipids..

[13] Spickett C.M., Pitt A.R. (2020). Modification of proteins by reactive lipid oxidation products and biochemical effects of lipoxidation. Essays Biochem..

[14] Guéraud F., Atalay M., Bresgen N., Cipak A., Eckl P.M., Huc L., Jouanin I., Siems W., Uchida K. (2010). Chemistry and biochemistry of lipid peroxidation products. Free Radic. Res..

[15] Zheng Y., Sun J., Luo Z., Li Y., Huang Y. (2024). Emerging mechanisms of lipid peroxidation in regulated cell death and its physiological implications. Cell Death Dis..

[16] Santin Y., Fazal L., Sainte-Marie Y., Sicard P., Maggiorani D., Tortosa F., Yücel Y.Y., Teyssedre L., Rouquette J., Marcellin M. (2020). Mitochondrial 4-HNE derived from MAO-A promotes mitoCa^2+^ overload in chronic postischemic cardiac remodeling. Cell Death Differ..

[17] Guéraud F. (2017). 4-Hydroxynonenal metabolites and adducts in pre-carcinogenic conditions and cancer. Free Radic. Biol. Med..

[18] Allowitz K., Taylor J., Harames K., Yoo J., Baloch O., Ramana K.V. (2025). Oxidative Stress-mediated Lipid Peroxidation-derived Lipid Aldehydes in the Pathophysiology of Neurodegenerative Diseases. Curr. Neuropharmacol..

[19] Cindrić M., Čipak Gašparović A., Milković L., Bujak I.T., Mihaljević B., Žarković N., Žarković K. (2022). 4-Hydroxynonenal modulates blood-brain barrier permeability in vitro through changes in lipid composition and oxidative status of endothelial cells and astrocytes. Int. J. Mol. Sci..

[20] Feng J., Wang J., Wang Y., Huang X., Shao T., Deng X., Cao Y., Zhou M., Zhao C. (2022). Oxidative Stress and Lipid Peroxidation: Prospective Associations Between Ferroptosis and Delayed Wound Healing in Diabetic Ulcers. Front. Cell Dev. Biol..

[21] Taso O.S., Philippou A., Moustogiannis A., Zevolis E., Koutsilieris M. (2019). Lipid peroxidation products and their role in neurodegenerative diseases. Ann. Res. Hosp..

[22] Stadtman E.R., Levine R.L. (2000). Protein oxidation. Ann. N. Y. Acad. Sci..

[23] Grimsrud P.A., Xie H., Griffin T.J., Bernlohr D.A. (2008). Oxidative stress and covalent modification of protein with bioactive aldehydes. J. Biol. Chem..

[24] Andrés C.M.C., Pérez de la Lastra J.M., Andrés Juan C., Plou F.J., Pérez-Lebeña E. (2022). Impact of Reactive Species on Amino Acids-Biological Relevance in Proteins and Induced Pathologies. Int. J. Mol. Sci..

[25] Koszła O., Sołek P. (2024). Misfolding and aggregation in neurodegenerative diseases: Protein quality control machinery as potential therapeutic clearance pathways. Cell Commun. Signal.

[26] Kim S., Kim E., Park M., Kim S.H., Kim B.G., Na S., Sadongo V.W., Wijesinghe W.C.B., Eom Y.G., Yoon G. (2024). Hidden route of protein damage through oxygen-confined photooxidation. Nat. Commun..

[27] Stringfellow H.M., Jones M.R., Green M.C., Wilson A.K., Francisco J.S. (2014). Selectivity in ROS-induced peptide backbone bond cleavage. J. Phys. Chem. A.

[28] Tramutola A., Falcucci S., Brocco U., Triani F., Lanzillotta C., Donati M., Panetta C., Luzi F., Iavarone F., Vincenzoni F. (2020). Protein Oxidative Damage in UV-Related Skin Cancer and Dysplastic Lesions Contributes to Neoplastic Promotion and Progression. Cancers.

[29] Rudan Dimlić M., Raić S., Močibob M., Sanader Maršić Ž., Yao Z., Radman M., Stagljar I. (2024). Oxidative protein damage negatively affects protein-protein interaction: The case of KRAS-cRAF. Biochem. Biophys. Res. Commun..

[30] Cecarini V., Gee J., Fioretti E., Amici M., Angeletti M., Eleuteri A.M., Keller J.N. (2007). Protein oxidation and cellular homeostasis: Emphasis on metabolism. Biochim. Biophys. Acta-Mol. Cell Res..

[31] Madkour L.H. (2020). Oxidative stress and oxidative damage-induced cell death. Reactive Oxygen Species (ROS), Nanoparticles, and Endoplasmic Reticulum (ER) Stress-Induced Cell Death Mechanisms.

[32] Yu Y., Cui Y., Niedernhofer L.J., Wang Y. (2016). Occurrence, Biological Consequences, and Human Health Relevance of Oxidative Stress-Induced DNA Damage. Chem. Res. Toxicol..

[33] Gonzalez-Hunt Claudia P., Mandheer Wadhwa Laurie H. (2018). Sanders, DNA damage by oxidative stress: Measurement strategies for two genomes. Curr. Opin. Toxicol..

[34] Hahm J.Y., Park J., Jang E.S., Chi S.W. (2022). 8-Oxoguanine: From oxidative damage to epigenetic and epitranscriptional modification. Exp. Mol. Med..

[35] Cooke M.S., Evans M.D., Dizdaroglu M., Lunec J. (2003). Oxidative DNA damage: Mechanisms, mutation, and disease. FASEB J..

[36] Kolbanovskiy M., Chowdhury M.A., Nadkarni A., Broyde S., Geacintov N.E., Scicchitano D.A., Shafirovich V. (2017). The Nonbulky DNA Lesions Spiroiminodihydantoin and 5-Guanidinohydantoin Significantly Block Human RNA Polymerase II Elongation In Vitro. Biochemistry.

[37] Dizdaroglu M., Coskun E., Jaruga P. (2017). Repair of oxidatively induced DNA damage by DNA glycosylases: Mechanisms of action, substrate specificities and excision kinetics. Mutat. Res./Rev. Mutat. Res..

[38] Caldecott K.W. (2024). Causes and consequences of DNA single-strand breaks. Trends Biochem. Sci..

[39] Li X., Yang C., Wu H., Chen H., Gao X., Zhou S., Zhang T.C., Ma W. (2024). DSB-induced oxidative stress: Uncovering crosstalk between DNA damage response and cellular metabolism. DNA Repair.

[40] Hegde M.L., Izumi T., Mitra S. (2012). Chapter 6—Oxidized base damage and single-strand break repair in mammalian genomes: Role of disordered regions and posttranslational modifications in early enzymes. Prog. Mol. Biol. Transl. Sci..

[41] Le Guen T., Ragu S., Guirouilh-Barbat J., Lopez B.S. (2014). Role of the double-strand break repair pathway in the maintenance of genomic stability. Mol. Cell. Oncol..

[42] Poetsch A.R. (2020). The genomics of oxidative DNA damage, repair, and resulting mutagenesis. Comput. Struct. Biotechnol. J..

[43] Kryston T.B., Georgiev A.B., Pissis P., Georgakilas A.G. (2011). Role of oxidative stress and DNA damage in human carcinogenesis. Mutat. Res./Fundam. Mol. Mech. Mutagen..

[44] Singh A., Kukreti R., Saso L., Kukreti S. (2019). Oxidative Stress: A Key Modulator in Neurodegenerative Diseases. Molecules.

[45] Maldonado E., Morales-Pison S., Urbina F., Solari A. (2023). Aging Hallmarks and the Role of Oxidative Stress. Antioxidants.

[46] Guillaumet-Adkins A., Yañez Y., Peris-Diaz M.D., Calabria I., Palanca-Ballester C., Sandoval J. (2017). Epigenetics and Oxidative Stress in Aging. Oxidative Med. Cell. Longev..

[47] Di Meo S., Reed T.T., Venditti P., Victor V.M. (2016). Role of ROS and RNS Sources in Physiological and Pathological Conditions. Oxidative Med. Cell. Longev..

[48] Komic L., Kumric M., Komic J., Tomicic M., Kurir T.T., Grahovac M., Mornar M., Rusic D., Bukic J., Bozic J. (2025). The Hidden Price of Plenty: Oxidative Stress and Calorie-Induced Cardiometabolic Dysfunction. Life.

[49] Koves T.R., Ussher J.R., Noland R.C., Slentz D., Mosedale M., Ilkayeva O., Bain J., Stevens R., Dyck J.R., Newgard C.B. (2008). Mitochondrial overload and incomplete fatty acid oxidation contribute to skeletal muscle insulin resistance. Cell Metab..

[50] Sverdlov A.L., Elezaby A., Behring J.B., Bachschmid M.M., Luptak I., Tu V.H., Siwik D.A., Miller E.J., Liesa M., Shirihai O.S. (2015). High fat, high sucrose diet causes cardiac mitochondrial dysfunction due in part to oxidative post-translational modification of mitochondrial complex II. J. Mol. Cell. Cardiol..

[51] Miotto P.M., LeBlanc P.J., Holloway G.P. (2018). High-Fat Diet Causes Mitochondrial Dysfunction as a Result of Impaired ADP Sensitivity. Diabetes.

[52] Țocu G., Ștefănescu B.I., Stavăr Matei L., Țocu L. (2025). Phagocyte NADPH Oxidase NOX2-Derived Reactive Oxygen Species in Antimicrobial Defense: Mechanisms, Regulation, and Therapeutic Potential-A Narrative Review. Antioxidants.

[53] Reuter S., Gupta S.C., Chaturvedi M.M., Aggarwal B.B. (2010). Oxidative stress, inflammation, and cancer: How are they linked?. Free Radic. Biol. Med..

[54] Phaniendra A., Jestadi D.B., Periyasamy L. (2015). Free radicals: Properties, sources, targets, and their implication in various diseases. Indian J. Clin. Biochem..

[55] Sharifi-Rad M., Anil Kumar N.V., Zucca P., Varoni E.M., Dini L., Panzarini E., Rajkovic J., Tsouh Fokou P.V., Azzini E., Peluso I. (2020). Lifestyle, Oxidative Stress, and Antioxidants: Back and Forth in the Pathophysiology of Chronic Diseases. Front. Physiol..

[56] Tulchinsky T.H., Varavikova E.A., Cohen M.J., Tulchinsky T.H., Varavikova E.A., Cohen M.J. (2023). Chapter 9—Environmental and occupational health. The New Public Health.

[57] Marchitti S.A., Chen Y., Thompson D.C., Vasiliou V. (2011). Ultraviolet radiation: Cellular antioxidant response and the role of ocular aldehyde dehydrogenase enzymes. Eye Contact Lens.

[58] Cortat B., Garcia C.C.M., Quinet A., Schuch A.P., De Lima-Bessa K.M., Menck C.F.M. (2013). The relative roles of DNA damage induced by UVA irradiation in human cells. Photochem. Photobiol. Sci..

[59] Reisz J.A., Bansal N., Qian J., Zhao W., Furdui C.M. (2014). Effects of ionizing radiation on biological molecules–mechanisms of damage and emerging methods of detection. Antioxid. Redox Signal..

[60] Spitz D.R., Azzam E.I., Li J.J., Gius D. (2004). Metabolic oxidation/reduction reactions and cellular responses to ionizing radiation: A unifying concept in stress response biology. Cancer Metast. Rev..

[61] Spitz D.R., Hauer-Jensen M. (2014). Ionizing radiation-induced responses: Where free radical chemistry meets redox biology and medicine. Antioxid. Redox Signal..

[62] Zhang P., Yang P., Hong L., Cai X., Zeng Z., Wu X., Zeng H., Shao L. (2020). Oxidative Stress in Radiation-Induced Cardiotoxicity. Oxidative Med. Cell. Longev..

[63] Pizzino G., Bitto A., Interdonato M., Galfo F., Irrera N., Mecchio A., Pallio G., Ramistella V., De Luca F., Minutoli L. (2014). Oxidative stress and DNA repair and detoxification gene expression in adolescents exposed to heavy metals living in the Milazzo-Valle del Mela area (Sicily, Italy). Redox Biol..

[64] Rao X., Zhong J., Brook R.D., Rajagopalan S. (2018). Effect of Particulate Matter Air Pollution on Cardiovascular Oxidative Stress Pathways. Antioxid. Redox Signal..

[65] Abolhasani R., Araghi F., Tabary M., Aryannejad A., Mashinchi B., Robati R.M. (2021). The impact of air pollution on skin and related disorders: A comprehensive review. Dermatol. Ther..

[66] Cassera E., Ferrari E., Vignati D.A.L., Capucciati A. (2025). The interaction between metals and catecholamines: Oxidative stress, DNA damage, and implications for human health. Brain Res. Bull..

[67] Caliri A.W., Tommasi S., Besaratinia A. (2021). Relationships among smoking, oxidative stress, inflammation, macromolecular damage, and cancer. Mutat. Res./Rev. Mutat. Res..

[68] Valavanidis A., Vlachogianni T., Fiotakis K., Loridas S. (2013). Pulmonary oxidative stress, inflammation and cancer: Respirable particulate matter, fibrous dusts and ozone as major causes of lung carcinogenesis through reactive oxygen species mechanisms. Int. J. Environ. Res. Public Health.

[69] Seo Y.S., Park J.M., Kim J.H., Lee M.Y. (2023). Cigarette Smoke-Induced Reactive Oxygen Species Formation: A Concise Review. Antioxidants.

[70] Jiang S., Liu H., Li C. (2021). Dietary Regulation of Oxidative Stress in Chronic Metabolic Diseases. Foods.

[71] Furukawa S., Fujita T., Shimabukuro M., Iwaki M., Yamada Y., Nakajima Y., Nakayama O., Makishima M., Matsuda M., Shimomura I. (2004). Increased oxidative stress in obesity and its impact on metabolic syndrome. J. Clin. Investig..

[72] Safdar A., Hamadeh M.J., Kaczor J.J., Raha S., Debeer J., Tarnopolsky M.A. (2010). Aberrant mitochondrial homeostasis in the skeletal muscle of sedentary older adults. PLoS ONE.

[73] Sánchez-Rodríguez M.A., Zacarías-Flores M., Correa-Muñoz E., Arronte-Rosales A., Mendoza-Núñez V.M. (2021). Oxidative Stress Risk Is Increased with a Sedentary Lifestyle during Aging in Mexican Women. Oxidative Med. Cell. Longev..

[74] Liguori I., Russo G., Curcio F., Bulli G., Aran L., Della-Morte D., Gargiulo G., Testa G., Cacciatore F., Bonaduce D. (2018). Oxidative stress, aging, and diseases. Clin. Interv. Aging.

[75] López-Otín C., Blasco M.A., Partridge L., Serrano M., Kroemer G. (2023). Hallmarks of aging: An expanding universe. Cell.

[76] Xu X., Pang Y., Fan X. (2025). Mitochondria in oxidative stress, inflammation and aging: From mechanisms to therapeutic advances. Signal Transduct. Target. Ther..

[77] Passos J.F., Saretzki G., Ahmed S., Nelson G., Richter T., Peters H., Wappler I., Birket M.J., Harold G., Schaeuble K. (2007). Mitochondrial dysfunction accounts for the stochastic heterogeneity in telomere-dependent senescence. PLoS Biol..

[78] Li J., O W., Li W., Jiang Z.-G., Ghanbari H.A. (2013). Oxidative Stress and Neurodegenerative Disorders. Int. J. Mol. Sci..

[79] Chandrasekaran A., Idelchik M.D.P.S., Melendez J.A. (2017). Redox control of senescence and age-related disease. Redox Biol..

[80] Santos D.F., Simão S., Nóbrega C., Bragança J., Castelo-Branco P., Araújo I.M. (2024). ALFA Score Consortium. Oxidative stress and aging: Synergies for age related diseases. FEBS Lett..

[81] Reddy V.P. (2023). Oxidative Stress in Health and Disease. Biomedicines.

[82] Dubois-Deruy E., Peugnet V., Turkieh A., Pinet F. (2020). Oxidative Stress in Cardiovascular Diseases. Antioxidants.

[83] Amponsah-Offeh M., Diaba-Nuhoho P., Speier S., Morawietz H. (2023). Oxidative Stress, Antioxidants and Hypertension. Antioxidants.

[84] Higashi Y. (2022). Roles of Oxidative Stress and Inflammation in Vascular Endothelial Dysfunction-Related Disease. Antioxidants.

[85] Caturano A., Rocco M., Tagliaferri G., Piacevole A., Nilo D., Di Lorenzo G., Iadicicco I., Donnarumma M., Galiero R., Acierno C. (2025). Oxidative Stress and Cardiovascular Complications in Type 2 Diabetes: From Pathophysiology to Lifestyle Modifications. Antioxidants.

[86] Kasai S., Kokubu D., Mizukami H., Itoh K. (2023). Mitochondrial Reactive Oxygen Species, Insulin Resistance, and Nrf2-Mediated Oxidative Stress Response-Toward an Actionable Strategy for Anti-Aging. Biomolecules.

[87] Guo C., Sun L., Chen X., Zhang D. (2013). Oxidative stress, mitochondrial damage and neurodegenerative diseases. Neural Regen. Res..

[88] Buga A.M., Oancea C.N. (2023). Oxidative Stress-Induced Neurodegeneration and Antioxidative Strategies: Current Stage and Future Perspectives. Antioxidants.

[89] Olufunmilayo E.O., Gerke-Duncan M.B., Holsinger R.M.D. (2023). Oxidative Stress and Antioxidants in Neurodegenerative Disorders. Antioxidants.

[90] Cheignon C., Tomas M., Bonnefont-Rousselot D., Faller P., Hureau C., Collin F. (2018). Oxidative stress and the amyloid beta peptide in Alzheimer’s disease. Redox Biol..

[91] Dhapola R., Beura S.K., Sharma P., Singh S.K., HariKrishnaReddy D. (2024). Oxidative stress in Alzheimer’s disease: Current knowledge of signaling pathways and therapeutics. Mol. Biol. Rep..

[92] Chakrabarti S., Bisaglia M. (2023). Oxidative Stress and Neuroinflammation in Parkinson’s Disease: The Role of Dopamine Oxidation Products. Antioxidants.

[93] Gilgun-Sherki Y., Melamed E., Offen D. (2004). The role of oxidative stress in the pathogenesis of multiple sclerosis: The need for effective antioxidant therapy. J. Neurol..

[94] Michel T.M., Pülschen D., Thome J. (2012). The role of oxidative stress in depressive disorders. Curr. Pharm. Des..

[95] Lei W., Zhuang H., Huang W., Sun J. (2025). Neuroinflammation and energy metabolism: A dual perspective on ischemic stroke. J. Transl. Med..

[96] Sahiner U.M., Birben E., Erzurum S., Sackesen C., Kalayci O. (2011). Oxidative stress in asthma. World Allergy Organ. J..

[97] Barnes P.J. (2022). Oxidative Stress in Chronic Obstructive Pulmonary Disease. Antioxidants.

[98] Da Fonseca L.J.S., Nunes-Souza V., Goulart M.O.F., Rabelo L.A. (2019). Oxidative Stress in Rheumatoid Arthritis: What the Future Might Hold regarding Novel Biomarkers and Add-On Therapies. Oxidative Med. Cell. Longev..

[99] Daenen K., Andries A., Mekahli D., Van Schepdael A., Jouret F., Bammens B. (2019). Oxidative stress in chronic kidney disease. Pediatr. Nephrol..

[100] Moloney J.N., Cotter T.G. (2018). ROS signalling in the biology of cancer. Semin. Cell Dev. Biol..

[101] Hayes J.D., Dinkova-Kostova A.T., Tew K.D. (2020). Oxidative Stress in Cancer. Cancer Cell..

[102] Jelic M.D., Mandic A.D., Maricic S.M., Srdjenovic B.U. (2021). Oxidative stress and its role in cancer. J. Cancer Res. Ther..

[103] Muguruma M., Unami A., Kanki M., Kuroiwa Y., Nishimura J., Dewa Y., Umemura T., Oishi Y., Mitsumori K. (2007). Possible involvement of oxidative stress in piperonyl butoxide induced hepatocarcinogenesis in rats. Toxicology.

[104] Morry J., Ngamcherdtrakul W., Yantasee W. (2017). Oxidative stress in cancer and fibrosis: Opportunity for therapeutic intervention with antioxidant compounds, enzymes, and nanoparticles. Redox Biol..

[105] Stanicka J., Russell E.G., Woolley J.F., Cotter T.G. (2015). NADPH oxidase-generated hydrogen peroxide induces DNA damage in mutant FLT3-expressing leukemia cells. J. Biol. Chem..

[106] Iqbal M.J., Kabeer A., Abbas Z., Siddiqui H.A., Calina D., Sharifi-Rad J., Cho W.C. (2024). Interplay of oxidative stress, cellular communication and signaling pathways in cancer. Cell Commun. Signal..

[107] Bellezza I., Giambanco I., Minelli A., Donato R. (2018). Nrf2-Keap1 signaling in oxidative and reductive stress. Biochim. Biophys. Acta (BBA)-Mol. Cell Res..

[108] Shi T., Dansen T.B. (2020). Reactive oxygen species induced p53 activation: DNA damage, redox signaling, or both?. Antioxid. Redox Signal..

[109] Cristofaro M., Contursi A., D’Amore S., Martelli N., Spaziante A.F., Moschetta A., Villani G. (2015). Adenomatous polyposis coli (APC)-induced apoptosis of HT29 colorectal cancer cells depends on mitochondrial oxidative metabolism. Biochim. Biophys. Acta (BBA)-Mol. Cell Res..

[110] Villeneuve N.F., Sun Z., Chen W., Zhang D.D. (2009). Nrf2 and p21 regulate the fine balance between life and death by controlling ROS levels. Cell Cycle.

[111] Long A.G., Lundsmith E.T., Hamilton K.E. (2017). Inflammation and Colorectal Cancer. Curr. Color. Cancer Rep..

[112] Verdina A., Garufi A., D’Orazi V., D’Orazi G. (2024). HIPK2 in Colon Cancer: A Potential Biomarker for Tumor Progression and Response to Therapies. Int. J. Mol. Sci..

[113] Hussain S.P., Harris C.C. (2007). Inflammation and cancer: An ancient link with novel potentials. Int. J. Cancer.

[114] Fernandes J.V., Cobucci R.N., Jatobá C.A., Fernandes T.A., de Azevedo J.W., de Araújo J.M. (2015). The role of the mediators of inflammation in cancer development. Pathol. Oncol. Res..

[115] Muthusami S., Ramachandran I.K., Babu K.N., Krishnamoorthy S., Guruswamy A., Queimado L., Chaudhuri G., Ramachandran I. (2021). Role of inflammation in the development of colorectal cancer. Endocr. Metab. Immune Disord.-Drug Targets.

[116] Wang M., Xiao Y., Miao J., Zhang X., Liu M., Zhu L., Liu H., Shen X., Wang J., Xie B. (2025). Oxidative Stress and Inflammation: Drivers of Tumorigenesis and Therapeutic Opportunities. Antioxidants.

[117] Kim Y.W., Byzova T.V. (2014). Oxidative stress in angiogenesis and vascular disease. Blood.

[118] Harrison I.P., Vinh A., Johnson I.R.D., Luong R., Drummond G.R., Sobey C.G., Tiganis T., Williams E.D., O’ Leary J.J., Brooks D.A. (2018). NOX2 oxidase expressed in endosomes promotes cell proliferation and prostate tumour development. Oncotarget.

[119] Cheng J., Yang H.L., Gu C.J., Liu Y.K., Shao J., Zhu R., He Y.Y., Zhu X.Y., Li M.Q. (2019). Melatonin restricts the viability and angiogenesis of vascular endothelial cells by suppressing HIF-1α/ROS/VEGF. Int. J. Mol. Med..

[120] Zhou N.J., Bao W.Q., Zhang C.F., Jiang M.L., Liang T.L., Ma G.Y., Liu L., Pan H.D., Li R.Z. (2025). Immunometabolism and oxidative stress: Roles and therapeutic strategies in cancer and aging. npj Aging.

[121] Tanaka A., Sakaguchi S. (2017). Regulatory T cells in cancer immunotherapy. Cell Res..

[122] Alissafi T., Kalafati L., Lazari M., Filia A., Kloukina I., Manifava M., Lim J.H., Alexaki V.I., Ktistakis N.T., Doskas T. (2020). Mitochondrial oxidative damage underlies regulatory T cell defects in autoimmunity. Cell Metab..

[123] Guo Z., Wang G., Wu B., Chou W.C., Cheng L., Zhou C., Lou J., Wu D., Su L., Zheng J. (2020). DCAF1 regulates Treg senescence via the ROS axis during immunological aging. J. Clin. Investig..

[124] Cheng A.N., Cheng L.C., Kuo C.L., Lo Y.K., Chou H.Y., Chen C.H., Wang Y.H., Chuang T.H., Cheng S.J., Lee A.Y. (2020). Mitochondrial Lon-induced mtDNA leakage contributes to PD-L1-mediated immunoescape via STING-IFN signaling and extracellular vesicles. J. Immunother. Cancer.

[125] Garg A.D., Dudek A.M., Ferreira G.B., Verfaillie T., Vandenabeele P., Krysko D.V., Mathieu C., Agostinis P. (2013). ROS-induced autophagy in cancer cells assists in evasion from determinants of immunogenic cell death. Autophagy.

[126] Benhar M. (2020). Oxidants, Antioxidants and Thiol Redox Switches in the Control of Regulated Cell Death Pathways. Antioxidants.

[127] Porté S., Valencia E., Yakovtseva E.A., Borràs E., Shafqat N., Debreczeny J.É., Pike A.C.W., Oppermann U., Farrés J., Fita I. (2009). Three-dimensional structure and enzymatic function of proapoptotic human p53-inducible quinone oxidoreductase PIG3. J. Biol. Chem..

[128] Redza-Dutordoir M., Averill-Bates D.A. (2016). Activation of apoptosis signalling pathways by reactive oxygen species. Biochim. Biophys. Acta (BBA)-Mol. Cell Res..

[129] Dixon S.J., Lemberg K.M., Lamprecht M.R., Skouta R., Zaitsev E.M., Gleason C.E., Patel D.N., Bauer A.J., Cantley A.M., Yang W.S. (2012). Ferroptosis: An iron-dependent form of nonapoptotic cell death. Cell.

[130] Yang W.S., SriRamaratnam R., Welsch M.E., Shimada K., Skouta R., Viswanathan V.S., Cheah J.H., Clemons P.A., Shamji A.F., Clish C.B. (2014). Regulation of ferroptotic cancer cell death by GPX4. Cell.

[131] Piskounova E., Agathocleous M., Murphy M.M., Hu Z., Huddlestun S.E., Zhao Z., Leitch A.M., Johnson T.M., DeBerardinis R.J., Morrison S.J. (2015). Oxidative stress inhibits distant metastasis by human melanoma cells. Nature.

[132] Kurutas E.B. (2016). The importance of antioxidants which play the role in cellular response against oxidative/nitrosative stress: Current state. Nutr. J..

[133] Chaudhary P., Janmeda P., Docea A.O., Yeskaliyeva B., Abdull Razis A.F., Modu B., Calina D., Sharifi-Rad J. (2023). Oxidative stress, free radicals and antioxidants: Potential crosstalk in the pathophysiology of human diseases. Front. Chem..

[134] Valko M., Leibfritz D., Moncol J., Cronin M.T., Mazur M., Telser J. (2007). Free radicals and antioxidants in normal physiological functions and human disease. Int. J. Biochem. Cell Biol..

[135] Rizzo A.M., Berselli P., Zava S., Montorfano G., Negroni M., Corsetto P., Berra B. (2010). Endogenous antioxidants and radical scavengers. Advances in Experimental Medicine and Biology.

[136] Rahaman M.M., Hossain R., Herrera-Bravo J., Islam M.T., Atolani O., Adeyemi O.S., Owolodun O.A., Kambizi L., Daştan S.D., Calina D. (2023). Natural antioxidants from some fruits, seeds, foods, natural products, and associated health benefits: An update. Food Sci. Nutr..

[137] Traber M.G., Stevens J.F. (2011). Vitamins C and E: Beneficial effects from a mechanistic perspective. Free Radic. Biol. Med..

[138] Caruso F., Pedersen J.Z., Incerpi S., Belli S., Sakib R., Rossi M. (2024). Interaction between Vitamins C and E When Scavenging the Superoxide Radical Shown by Hydrodynamic Voltammetry and DFT. Biophysica.

[139] Upadhyay S., Dixit M. (2015). Role of Polyphenols and Other Phytochemicals on Molecular Signaling. Oxidative Med. Cell. Longev..

[140] Bhuyan U., Handique J.G., Attaur-Rahman (2022). Chapter 6—Plant polyphenols as potent antioxidants: Highlighting the mechanism of antioxidant activity and synthesis/development of some polyphenol conjugates. Studies in Natural Products Chemistry.

[141] Fiedor J., Burda K. (2014). Potential role of carotenoids as antioxidants in human health and disease. Nutrients.

[142] Adwas A.A., Elsayed A., Azab A.E., Quwaydir F.A. (2019). Oxidative stress and antioxidant mechanisms in human body. J. Appl. Biotechnol. Bioeng..

[143] Sotler R., Poljšak B., Dahmane R., Jukić T., Pavan Jukić D., Rotim C., Trebše P., Starc A. (2019). Prooxidant activities of antioxidants and their impact on health. Acta Clin. Croat..

[144] Kaźmierczak-Barańska J., Boguszewska K., Adamus-Grabicka A., Karwowski B.T. (2020). Two Faces of Vitamin C-Antioxidative and Pro-Oxidative Agent. Nutrients.

[145] Maliar T., Maliarová M., Blažková M., Kunštek M., Uváčková Ľ., Viskupičová J., Purdešová A., Beňovič P. (2023). Simultaneously Determined Antioxidant and Pro-Oxidant Activity of Randomly Selected Plant Secondary Metabolites and Plant Extracts. Molecules.

[146] Yin J.J., Fu P.P., Lutterodt H., Zhou Y.T., Antholine W.E., Wamer W. (2012). Dual role of se-lected antioxidants found in dietary supplements: Crossover between anti- and pro-oxidant activities in the presence of copper. J. Agric. Food Chem..

[147] Dzah C., Zhang H., Gobe V., Asante-Donyinah D., Yuqing D. (2024). Anti- and pro-oxidant properties of polyphenols and their role in modulating glutathione synthesis, activity and cellular redox potential: Potential synergies for disease management. Adv. Redox Res..

[148] Barbouti A., Goulas V. (2021). Dietary Antioxidants in the Mediterranean Diet. Antioxidants.

[149] Aleksandrova K., Koelman L., Rodrigues C.E. (2021). Dietary patterns and biomarkers of oxidative stress and inflammation: A systematic review of observational and intervention studies. Redox Biol..

[150] Nouri M., Soltani M., Rajabzadeh-Dehkordi M., Rafieipour N., Askarpour M., Najafi M., Faghih S. (2023). Dietary Antioxidant Capacity Indices are Negatively Correlated to LDL-Oxidation in Adults. Int. J. Clin. Pract..

[151] Urquiaga I., Strobel P., Perez D., Martinez C., Cuevas A., Castillo O., Marshall G., Rozowski J., Leighton F. (2010). Mediterranean diet and red wine protect against oxidative damage in young volunteers. Atherosclerosis.

[152] Billingsley H.E., Carbone S. (2018). The antioxidant potential of the Mediterranean diet in patients at high cardiovascular risk: An in-depth review of the PREDIMED. Nutr. Diabetes.

[153] Pirouzeh R., Heidarzadeh-Esfahani N., Morvaridzadeh M., Izadi A., Yosaee S., Potter E., Heshmati J., Pizarro A.B., Omidi A., Heshmati S. (2020). Effect of DASH diet on oxidative stress parameters: A systematic review and meta-analysis of randomized clinical trials. Diabetes Metab. Syndr..

[154] Arab A., Khorvash F., Karimi E., Heidari Z., Askari G. (2022). The effects of the dietary approaches to stop hypertension (DASH) diet on oxidative stress and clinical indices of migraine patients: A randomized controlled trial. Nutr. Neurosci..

[155] Issaoui M., Delgado A.M., Caruso G., Micali M., Barbera M., Atrous H., Ouslati A., Chammem N. (2020). Phenols, Flavors, and the Mediterranean Diet. J. AOAC Int..

[156] Hossain M.S., Wazed M.A., Asha S., Amin M.R., Shimul I.M. (2025). Dietary Phytochemicals in Health and Disease: Mechanisms, Clinical Evidence, and Applications-A Comprehensive Review. Food Sci. Nutr..

[157] Cuciniello R., Di Meo F., Filosa S., Crispi S., Bergamo P. (2023). The Antioxidant Effect of Dietary Bioactives Arises from the Interplay between the Physiology of the Host and the Gut Microbiota: Involvement of Short-Chain Fatty Acids. Antioxidants.

[158] Heshmati J., Morvaridzadeh M., Maroufizadeh S., Akbari A., Yavari M., Amirinejad A., Maleki-Hajiagha A., Sepidarkish M. (2019). Omega-3 fatty acids supplementation and oxidative stress parameters: A systematic review and meta-analysis of clinical trials. Pharmacol. Res..

[159] Jiang Y., Zhao Y., Milne G., Dai Q., Chen Q., Zhang X., Lan Q., Rothman N., Gao Y.T., Cai Q. (2022). Quality of dietary carbohydrate is more important than its quantity in lipid peroxidation. Am. J. Clin. Nutr..

[160] Carlson D.A., True C., Wilson C.G. (2024). Oxidative stress and food as medicine. Front. Nutr..

[161] Yang M., Zhou X., Tan X., Huang X., Yuan L., Zhang Z., Yang Y., Xu M., Wan Y., Li Z. (2022). The Status of Oxidative Stress in Patients with Alcohol Dependence: A Meta-Analysis. Antioxidants.

[162] Bosch-Sierra N., Grau-del Valle C., Salom C., Zaragoza-Villena B., Perea-Galera L., Falcón-Tapiador R., Rovira-Llopis S., Morillas C., Monleón D., Bañuls C. (2024). Effect of a Very Low-Calorie Diet on Oxidative Stress, Inflammatory and Metabolomic Profile in Metabolically Healthy and Unhealthy Obese Subjects. Antioxidants.

[163] Ursini F., Sevanian A. (2002). Postprandial oxidative stress. Biol. Chem..

[164] Bae J.H., Bassenge E., Kim K.B., Kim Y.N., Kim K.S., Lee H.J., Moon K.C., Lee M.S., Park K.Y., Schwemmer M. (2001). Postprandial hypertriglyceridemia impairs endothelial function by enhanced oxidant stress. Atherosclerosis.

[165] Clemente-Suárez V.J., Beltrán-Velasco A.I., Redondo-Flórez L., Martín-Rodríguez A., Tornero-Aguilera J.F. (2023). Global Impacts of Western Diet and Its Effects on Metabolism and Health: A Narrative Review. Nutrients.

[166] Bakker S.J., IJzerman R.G., Teerlink T., Westerhoff H.V., Gans R.O., Heine R.J. (2000). Cytosolic triglycerides and oxidative stress in central obesity: The missing link between excessive atherosclerosis, endothelial dysfunction, and beta-cell failure?. Atherosclerosis.

[167] Tsai W.C., Li Y.H., Lin C.C., Chao T.H., Chen J.H. (2004). Effects of oxidative stress on endothelial function after a high-fat meal. Clin. Sci..

[168] Dierckx N., Horvath G., van Gils C., Vertommen J., van de Vliet J., De Leeuw I., Manuel-y-Keenoy B. (2003). Oxidative stress status in patients with diabetes mellitus: Relationship to diet. Eur. J. Clin. Nutr..

[169] Poznyak A.V., Nikiforov N.G., Markin A.M., Kashirskikh D.A., Myasoedova V.A., Gerasimova E.V., Orekhov A.N. (2021). Overview of OxLDL and Its Impact on Cardiovascular Health: Focus on Atherosclerosis. Front. Pharmacol..

[170] Hanssen N.M.J., Kraakman M.J., Flynn M.C., Nagareddy P.R., Schalkwijk C.G., Murphy A.J. (2020). Postprandial Glucose Spikes, an Important Contributor to Cardiovascular Disease in Diabetes?. Front. Cardiovasc. Med..

[171] Sies H., Stahl W., Sevanian A. (2005). Nutritional, dietary and postprandial oxidative stress. J. Nutr..

[172] Ansar S., Koska J., Reaven P.D. (2011). Postprandial hyperlipidemia, endothelial dysfunction and cardiovascular risk: Focus on incretins. Cardiovasc. Diabetol..

[173] Vogel R.A., Corretti M.C., Plotnick G.D. (1997). Effect of a single high-fat meal on endothelial function in healthy subjects. Am. J. Cardiol..

[174] Fewkes J.J., Kellow N.J., Cowan S.F., Williamson G., Dordevic A.L. (2022). A single, high-fat meal adversely affects postprandial endothelial function: A systematic review and meta-analysis. Am. J. Clin. Nutr..

[175] Choleva M., Matalliotaki E., Antoniou S., Asimomyti E., Drouka A., Stefani M., Yannakoulia M., Fragopoulou E. (2022). Postprandial Metabolic and Oxidative Stress Responses to Grape Pomace Extract in Healthy Normal and Overweight/Obese Women: A Randomized, Double-Blind, Placebo-Controlled Crossover Study. Nutrients.

[176] Baynham R., Veldhuijzen van Zanten J.J.C.S., Rendeiro C. (2024). Cocoa flavanols rescue stress-induced declines in endothelial function after a high-fat meal, but do not affect cerebral oxygenation during stress in young, healthy adults. Food Funct..

[177] Ceriello A., Bortolotti N., Motz E., Lizzio S., Catone B., Assaloni R., Tonutti L., Taboga C. (2001). Red wine protects diabetic patients from meal-induced oxidative stress and thrombosis activation: A pleasant approach to the prevention of cardiovascular disease in diabetes. Eur. J. Clin. Investig..

[178] Grassi D., Mai F., De Feo M., Barnabei R., Carducci A., Desideri G., Necozione S., Allegaert L., Bernaert H., Ferri C. (2023). Cocoa Consumption Decreases Oxidative Stress, Proinflammatory Mediators and Lipid Peroxidation in Healthy Subjects: A Randomized Placebo-Controlled Dose-Response Clinical Trial. High Blood Press. Cardiovasc. Prev..

